# M. Lisfranc on Extirpation of Part of the Rectum

**Published:** 1830-07-01

**Authors:** 


### M. LISFRANC ON EXTIRPATION OF THE LOWER PORTION OF THE RECTUM FOR CANCEROUS DISEASE.*

We believe it is well known by most of our readers, that M. Lisfranc is considered the Coryphæus of the excisors of the uterus, or parts of it. Numerous successful cases of extirpation of scirrhous cervix uteri have been published by that gentleman, but the extreme laxity of the French in the employment of the terms scirrhus and cancer, has rendered the actual nature of many of M. Lisfranc's cases more than doubtful. The same surgeon has recently read to the members of the Royal Academy of France, a memoir on Excision of the lower Portion of the Rectum affected with Malignant Disease, and of this a good resumé has been published in a valuable Parisian contemporary.

In a proper case for the operation, the fore-finger, introduced into the intestine, should admit of being carried beyond the limits of the disease, and the operator must also have determined the thickness of the scirrhous gut. If not only the parietes of the latter, but the neighbouring cellular membrane be affected, M. Lisfranc doubts of the propriety of operating, having once been embarrassed in this manner himself. Prior to the operation, the bowels should be emptied as far as possible of fæcal matters, and the patient should be placed in the position for lithotomy. We now proceed to the steps of the operation.

The extremity of the rectum should be included between two semi-lunar incisions of the skin, meeting an inch before and the same behind the anus. The lower portion is then to be separated from the neighbouring parts by dissecting away the skin, &c. and the rectum brought down lower by means of the fore-finger in its interior. When the disease is superficial, not involving more than the mucous membrane or confined to the tunics of the intestine, and when it extends no higher than an inch, the whole may be readily brought into view, and removed by a pair of scissors curved on their flat plane.

When, however, the disease has extended to the cellular membrane, and reaches as high as two or three inches, an incision must be made in the gut with strong straight scissors (after the preliminary steps described) parallel to its axis, in the posterior part, and it should be extended above the limits of the disease. Two or three hooks are then to be fixed in the inferior part of the rectum, in order to depress it, and if the patient be a female, an assistant, renders the recto-vaginal paries prominent by means of two fingers in the vagina; if a male, the urethra is projected by a catheter introduced into the canal. These precautions being taken, the dissection of the anterior part of the rectum is slowly and cautiously effected, and that being done, the separation of the remainder is more easy. The operation, when the cellular tissue is affected, is more difficult in man than in woman, on account of the comparative smallness of his pelvis, the interference of the prostate, &c. but it is by no means unattended with embarrassment in the other sex. The vessels are tied as they are opened, and M. Lisfranc has generally found them small, nor does he think that the internal pudic can be wounded. When the lower part of the gut is excised, the remainder escapes so high as to render it difficult at times to seize it with the fingers and draw it down.

After the operation, M. Lisfranc allows a moderate hæmorrhage to take place, taking care that the patient is not threatened with syncope. The

* Journal de Progrés, tome I. 1830.

wound is dressed with simple ointment, lint, compresses, and T bandage. When the patient is a female, her urine should be drawn off for the first 15 or 20 days, in order to prevent its irritating the wound. Copious suppuration ensues in the latter, and does not begin to diminish till the expiration of three or four weeks. Cicatrization is at first rapid but subsequently slower, and as soon as the opening begins to contract, a large plug of lint is to be introduced on the forefinger, taking care that it enters properly the cavity of the intestine. In none of the patients who were cured has the power of retaining the fæces been lost, for a kind of pad (bourrelet) forms at the extremity of the intestine, and operates as a sphincter. The more of the rectum that has been removed, the higher is the *bourrelet*, and the greater the tendency of the fæces to lodge about the part. The patient is constrained, under these circumstances, to remove them with his finger, "a trifling inconvenience," and one individual was obliged to keep a plug of lint in the aperture, and only remove it when passing a motion. Hæmorrhage to any extent is a rare occurrence, but if syncope is threatened, the rectum must be plugged for three or four hours, if the vessel cannot be secured. A set of nervous symptoms, such as shiverings, hiccup, vomiting, and frequent desire to stool, very frequently follow the operation, and the vomiting, especially, may continue for several days. M. Lisfranc has performed the operation on nine individuals, six of whom were females; two women and one man died, or one in three. Two dissections were made, one shewing infiltration of pus in the pelvis, the other inflammation of the veins. We shall give one successful case as a sample of the whole.

*Case.* Lise Fauniere, æt. 25, entered the Pitié on the 10th June, 1828. She had led a most infamous life, suffered pains in the inferior part of the rectum for three years, and been submitted to anti-syphilitic treatment at the Venereal Hospital. On admission her aspect was squalid, the stools were passed with pain and tenesmus, mixed with blood, and in very small quantity. On introducing the finger into the gut, a scirrhous thickening of the parietes, reaching to three inches and a half from the anus, was distinguished, and the finger with difficulty traversed the contracted passage. M. Lisfranc operated on the patient, Aug. 17th, 1828, in the manner already described. Only one artery required ligature, a sponge filled with water was placed in the wound, and in two hours all oozing of blood had ceased. At 6, p.m. the pulse having risen, a free bleeding from the arm was had recourse to, and the patient obtained a good night's rest. The dressings becoming very foul, and the wound very painful, from the contact of the urine and fæces, the water was drawn off by the catheter, the wound cleaned out by an injection of infusion of mallows, and two more bleedings from the arm employed. On the 20th, the secretion of very fetid pus commenced, and, on the 26th, the suppuration was become abundant. The wound was dressed twice a-day, and on the 10th of September it had diminished in extent, the suppuration was less abundant, and the lower part was beginning to contract. A large plug was introduced into the inferior part of the gut, and on the 20th, the wound was reduced to the size of a five-franc piece. The *bourrelet* was now formed in part, and under the application of the nitrate of silver to redundant granulations, and the introduction of a large plug dipped in the chloride of sodium, the wound was quite healed on the 25th Nov. In consequence of the tendency to contraction of the passage, the introduction of plugs was continued, and the patient remained in the hospital till the 28th of April, 1829, in order to ascertain the results of the operation. She retained her fæces well, and was able herself to introduce plugs of lint from time to time, in order to maintain the calibre of the canal. This patient was presented to the Academy, and still continues cured.

We do not imagine that this operation will ever be so common in this country as it is in the hands of M. Lisfranc. The operation itself is difficult, in some cases impossible, and dangerous in all, as the result of M. Lisfranc's operations proves. If the disease be really cancer the chances of a cure are problematical, the difficulties, pain, and danger certain; and if it be not of a malignant nature, no surgeon would venture to perform or to propose such an operation. But such questions as these are not so easily settled, for although the operation be of questionable propriety in one extreme case, and decidedly objectionable in the other, there are some affections of the rectum which are not sufficiently malignant to contaminate the constitution to an irreparable extent, nor sufficiently benignant to admit of any remedy short of a total removal. Whether M. Lisfranc's operation be adapted to such cases is a point which we leave to our able surgical brethren.

